# Widespread recovery of methylation at gametic imprints in hypomethylated mouse stem cells following rescue with DNMT3A2

**DOI:** 10.1186/s13072-016-0104-2

**Published:** 2016-11-22

**Authors:** Avinash Thakur, Sarah-Jayne Mackin, Rachelle E. Irwin, Karla M. O’Neill, Gareth Pollin, Colum Walsh

**Affiliations:** 1 Genomic Medicine Research Group, Biomedical Sciences Research Institute, Centre for Molecular Biosciences, University of Ulster, Coleraine, BT52 1SA UK; 2 Terry Fox Laboratory, BC Cancer Agency, 675 W 10th Ave, Vancouver, BC V5Z 1G1 Canada; 3 Centre for Experimental Medicine, The Wellcome-Wolfson Institute for Experimental Medicine, Queen’s University Belfast, 97 Lisburn Road, Belfast, BT9 7AE UK

**Keywords:** Imprinting, DNA methylation, Reprogramming, ESC

## Abstract

**Background:**

Imprinted loci are paradigms of epigenetic regulation and are associated with a number of genetic disorders in human. A key characteristic of imprints is the presence of a gametic differentially methylated region (gDMR). Previous studies have indicated that DNA methylation lost from gDMRs could not be restored by DNMT1, or the de novo enzymes DNMT3A or 3B in stem cells, indicating that imprinted regions must instead undergo passage through the germline for reprogramming. However, previous studies were non-quantitative, were unclear on the requirement for DNMT3A/B and showed some inconsistencies. In addition, new putative gDMR has recently been described, along with an improved delineation of the existing gDMR locations. We therefore aimed to re-examine the dependence of methylation at gDMRs on the activities of the methyltransferases in mouse embryonic stem cells (ESCs).

**Results:**

We examined the most complete current set of imprinted gDMRs that could be assessed using quantitative pyrosequencing assays in two types of ESCs: those lacking DNMT1 (1KO) and cells lacking a combination of DNMT3A and DNMT3B (3abKO). We further verified results using clonal analysis and combined bisulfite and restriction analysis. Our results showed that loss of methylation was approximately equivalent in both cell types. 1KO cells rescued with a cDNA-expressing DNMT1 could not restore methylation at the imprinted gDMRs, confirming some previous observations. However, nearly all gDMRs were remethylated in 3abKO cells rescued with a DNMT3A2 expression construct (3abKO + 3a2). Transcriptional activity at the *H19/Igf2* locus also tracked with the methylation pattern, confirming functional reprogramming in the latter.

**Conclusions:**

These results suggested (1) a vital role for DNMT3A/B in methylation maintenance at imprints, (2) that loss of DNMT1 and DNMT3A/B had equivalent effects, (3) that rescue with DNMT3A2 can restore imprints in these cells. This may provide a useful system in which to explore factors influencing imprint reprogramming.

**Electronic supplementary material:**

The online version of this article (doi:10.1186/s13072-016-0104-2) contains supplementary material, which is available to authorized users.

## Background

In mouse, DNA methylation is found predominantly at cytosine when followed by guanine (CpG) and is associated with various biological functions including the regulation of gene expression, X chromosome inactivation, silencing of retrotransposons and imprinting [1]. Many CpGs are protected from methylation by being clustered into CpG islands (CGI), which are commonly found near the transcriptional start sites of genes and are normally unmethylated, except for CGI on the inactive X or on inactive imprinted alleles. DNMT1, a maintenance methyltransferase [2], is crucial to ensure the regular propagation of DNA methylation patterns to the daughter strand during replication [3]. This enzyme is predominantly found near replication foci [4] and preferentially targets hemi-methylated DNA [4–6] suggesting its main functions as a maintenance methyltransferase [7–9]. The addition of methylation to an unmethylated template (de novo) is carried out by DNMT3A and DNMT3B, with the former responsible for most de novo activity in germ cells [10], while the latter predominates in somatic tissues [11]. However, in addition to their de novo methylation activity, several reports on DNMT3A and DNMT3B indicate a role in methylation maintenance in embryonic stem cells (ESCs), although the extent of their requirement at imprinted loci remains unclear [12, 13].

Once established on a DNA duplex, methylation is stably maintained through most of life [14, 15], but during certain developmental stages undergoes large-scale changes [11–13, 16]. Methylation patterns inherited from the sperm and oocyte are remodelled during pre-implantation development, when the paternal and maternal genomes of the embryo undergo widespread active demethylation involving the TET enzymes as well as passive demethylation via replicative dilution [15, 17]. The blastocyst stage sees methylation reach its nadir, but following implantation, a wave of de novo methylation occurs causing overall global hypermethylation at most non-island CpG in the adult tissues [18]. This de novo activity is present at high levels in ESCs [5], developing germ cells and early post-implantation embryos [19] but is present at lower levels in somatic cells [20, 21]. The presence of de novo activity in ESCs makes these cells a suitable model to study the mechanism of de novo methylation in mammals.

One group of genes that largely escapes global methylation remodelling during somatic development is the imprinted genes [14, 15]. These are a group of genes which exhibit expression from one parental allele only [22, 23]. Regulation of imprinting has biological significance as imprinted genes are important for embryonic development and their dysregulation leads to embryonic death in mouse and to various disease syndromes in human [22]. Initiation of allele-specific gene methylation patterns starts in the male and female germline during gametogenesis [24]. For imprinted genes, one of the parental alleles acquires DNA methylation at certain locations, and these are detected as differentially methylated regions (DMRs) in somatic cells [25]. Due to their origin in the germline, they are known as the gametic differentially methylated regions (gDMRs) [26], to distinguish them from other types of DMR such as tissue-specific DMR. Some of the gDMR are at *cis-*acting regulatory regions and are known to control monoallelic expression of more than one linked gene: where this has been proven by experimentation the DMRs are called imprinting control regions (ICRs) [27–31]. Methylation at gDMRs is established in the germline largely by the de novo methyltransferase DNMT3A with the aid of the essential cofactor DNMT3L [32–35]. The gDMRs at imprinted regions exhibit the property of being able to resist the processes of active and passive DNA demethylation during the pre-implantation stages of mammalian development or iPS formation [14, 18, 36, 37].

Loss of imprinting is thought to be irreversible and requires germline passage for its recovery due to the presence of essential factors and de novo methyltransferases needed for imprint establishment there [38]. Previous work has shown that rescuing DNA methyltransferase activity in *Dnmt1* ^−*/*−^ (1KO) cells by adding back a cDNA expressing the enzyme failed to restore methylation at paternal and maternal ICRs [38]. Other laboratories confirmed this but reported, however, that the paternally imprinted *H19* gDMR regained methylation in *Dnmt3a* ^−*/*−^; *Dnmt3b* ^−*/*−^ double knock-out (3abKO) cells rescued with a DNMT3A2 expression plasmid [39], suggesting that some imprints could be somatically reprogrammed. As well as these differing results, the early studies were carried out on a very limited number of gDMRs using qualitative approaches, which had limited resolution. Given the important implications somatic resetting could have for imprinted disease syndromes as well as cellular reprogramming generally, we wished to re-examine whether methylation at gDMRs could be established outside of the germline. Recent work has delineated the gDMRs far more sharply since the original studies were carried out, and more quantitative techniques are now available. We aimed to investigate (1) whether deletion of *Dnmt3ab* gives comparable methylation loss at imprinted loci to *Dnmt1* mutated cells; (2) whether imprints can be restored in 3abKO cells, unlike 1KO ESCs; (3) does loss of methylation result in dysregulated expression of imprinted genes; and (4) are there any exceptional imprinted gDMRs that do not regain methylation in rescued cells?

## Methods

### Statistical analysis

All laboratory experiments were carried out in triplicate with at least one biological repeat, with one or two exceptions as noted. Pyrosequencing, bisulfite sequencing and RT-qPCR data are represented as graphs, where error bars represent standard error of the mean (s.e.m). Statistical analysis was carried out using EXCEL and GraphPad PRISM software; for pyrosequencing data were compared by *t* test and Kruskal–Wallis, and bisulfite clonal analysis comparison was made using the *χ* ^2^ test.

### Cells

All cell culture media components were purchased from Invitrogen (Paisley, UK). *Dnmt1* KO and *Dnmt3a/3b* double KO cells with matching WT were kind gifts from Dr. Masaki Okano (RIKEN Center for Developmental Biology, Kobe, Japan). ESCs were maintained on Nunc plates (Davidson & Hardy, Belfast, UK) treated with 0.1% gelatin (Sigma-Aldrich, Dorset, UK) and cultured in Knockout DMEM plus 15% knockout serum replacement, 1% ESC-qualified Foetal Bovine Serum, 1× NEAA, 2 mM l-glutamine, 0.1 mM β-mercaptoethanol (Sigma-Aldrich, Dorset, UK) and 1000U/ml LIF (Merck Millipore, Hertfordshire, UK).

### Animal work

Tissues of interest were derived from outbred TO mice (Harlan, Huntingdon, UK). Sperm collection was carried out as previously described [13].

### RNA extraction, cDNA synthesis and RT-qPCR

RNA was extracted using the RNeasy kit (Qiagen, Crawley, UK), according to the manufacturer’s instructions. For cDNA synthesis, 300–500 ng RNA was used in combination with 0.5 μg random primers (Roche, West Sussex, UK), 40 U RNaseOUT 0.5 μM dNTPs (Invitrogen, Paisley, UK) 1× RT Buffer (Fermentas, Cambridge, UK) and RevertAid reverse transcriptase (Fermentas, Cambridge, UK) made up to a final volume of 20 μl using RNase-free water (Qiagen, Crawley, UK). Reactions were carried out in a thermocycler with conditions—25 °C for 10 min, 42 °C for 60 min and 70 °C for 10 min. One microlitre cDNA per well on a 96-well plate (Roche) was used for RT-qPCR with SYBR Green reagent and remaining cDNA stored at −80 °C. RT-qPCRs were performed using a LightCycler 480 Instrument II (Roche, West Sussex, UK). Gene expression was normalised to *Hprt* and relative expression calculated by the ΔΔC_*T*_ method [40]. Each RT-qPCR contained 1× buffer, 0.4 mM dNTPs, 50 μM primers (Additional file 1: Table S1), 0.01 U Taq DNA polymerase (Invitrogen, Paisley, UK) and nuclease-free water (Qiagen, Crawley, UK). Four primer sets for *Dnmt1*, *Dnmt3a*, *Dnmt3b* [47] and *Hprt* [13] were used. The general thermocycler conditions are as follows—94 °C for 3 min, followed by 30 cycles of 94 °C for 30 s, 63 °C for 1 min, 72 °C for 1 min with a final elongation step of 72 °C for 4 min.

#### Protein analysis

Protein was extracted from cells growing in log phase using protein extraction buffer (50 mM Tris–HCl, 150 mM NaCl, 1% Triton-X, 10% glycerol, 5 mM EDTA; all Sigma-Aldrich) and 0.5 µl protease inhibitor mix (Sigma-Aldrich, Dorset, UK). For Western blotting, 30 μg protein was denatured in the presence of 5 μl 4× LDS sample buffer (Invitrogen, Paisley, UK) and 2 μl 10× reducing agent (Invitrogen) in a total volume of 20 μl nuclease-free water (Qiagen, Crawley, UK) via incubation at 70 °C. Proteins were fractionated on a 4–12% SDS-PAGE gel, then electroblotted onto a nitrocellulose membrane (Invitrogen, Paisley, UK) and blocked in 5% non-fat milk for 1 h at room temperature (RT). Membranes were incubated with anti-DNMT1 (ab87654, Abcam), anti-DNMT3A (clone 64B1446, Novus Biologicals, Abingdon, UK), anti-GAPDH (clone 14C10, Cell Signalling Technologies, Leiden, Netherlands) or anti-β-actin (clone AC-15, Sigma-Aldrich) overnight at 4 °C, followed by incubation with the relevant HRP-conjugated secondary antibody (Sigma-Aldrich, Dorset, UK) for 1 h at RT and then visualised using ECL (ThermoFisher Scientific, Loughborough, UK).

### DNA isolation and bisulfite conversion

DNA extraction from sperm and tissues was as previously described [41]. All ESCs were pelleted and incubated overnight at 55 °C in lysis buffer (50 mM Tris pH 8, 0.1 M EDTA, 0.5% SDS (all from Sigma-Aldrich, Dorset, UK), 0.2 mg/ml proteinase K (Roche, West Sussex, UK) with rotation. DNA was extracted next day using the phenol/chloroform/isoamylalcohol (25:24:1, pH 8.0; Sigma-Aldrich, Dorset, UK) extraction method. The integrity of the DNA was checked on a 1% agarose gel (Eurogentec, Southampton, UK) and quality and quantity checked using a NanoDrop UV spectrophotometer (Labtech International, Ringmer, UK). For bisulfite conversion, 500 ng of DNA was processed with the EpiTect Bisulfite Kit (Qiagen, Crawley, UK) or EZ DNA Methylation Kit (Zymo, Cambridge, UK) according to manufacturer’s instructions.

### Methylation analysis

Bisulfite-converted DNA was PCR amplified in a reaction containing 1 μM primers, 1× buffer and 0.4 mM dNTPs, with MgCl_2_ at a concentration specific to the primer set and 0.01U *Taq* DNA polymerase (all reagents from Invitrogen, Paisley, UK). Combined bisulfite restriction analysis (COBRA) on genes was carried out as previously described [13] using *Taqα*I enzyme for *H19* and *KvDMR* and *BstU*I for *Snrpn* (both New England Biolabs, Hitchin, UK). Clonal analysis of bisulfite-converted PCR-amplified products in pJET1.2 vector (Fermentas, Cambridge, UK) was carried out using a PRISM 3130 Genetic Analyzer (Applied Biosystems, Paisley, UK). All pyrosequencing assays (Additional File 2: Table S2) were designed in-house using PyroMark (V2.0) assay design software (Qiagen, Crawley, UK). The PyroMark PCR Kit (Qiagen, Crawley, UK) was used to amplify genes using a thermocycler (Techne, Stone, UK) with conditions: 95 °C, 15 min; followed by 45 cycles of 94 °C for 30 s, 56 °C for 30 s and 72 °C for 30 s; with final elongation at 72 °C for 10 min. Subsequent pyrosequencing was carried out using Pyromark reagents as per manufacturer’s recommendations (Qiagen, Crawley, UK); 2 M NaOH was from Sigma-Aldrich (Dorset, UK) and Sepharose beads from GE Healthcare (Chalfont St. Giles, UK).

The Luminometric Methylation Assay (LUMA) using 300 ng/μl of genomic DNA from the respective cell lines was carried out exactly as described previously [13, 49]. HCT116 WT DNA (hypermethylated) and DKO DNA (hypomethylated) samples were used as a control (data not shown).

### Optimising primer alignment with galaxy user-defined tracks

Wang et al. [15] provided chromosomal coordinates for numerous known and putative germline imprints as part of their supplemental material. The coordinates delineated for each imprint were used as a tool to define the minimal gDMR regions, from which the respective genomic sequence was extracted by visualising these regions on UCSC genome browser. The extracted genomic sequence was used to promote specificity in the design of pyrosequencing assays. BED files were generated using these chromosomal coordinates and uploaded through the Galaxy interface [42] as user-defined tracks visible on UCSC genome browser. The genomic sequence of interest generated from each respective imprint primer set created was matched using the BLAT tool at UCSC against the user-defined track to confirm the positions of the assays (Additional file 1: Table S1).

## Results

### Initial gDMR examined and regions assayed

**Fig. 1:**
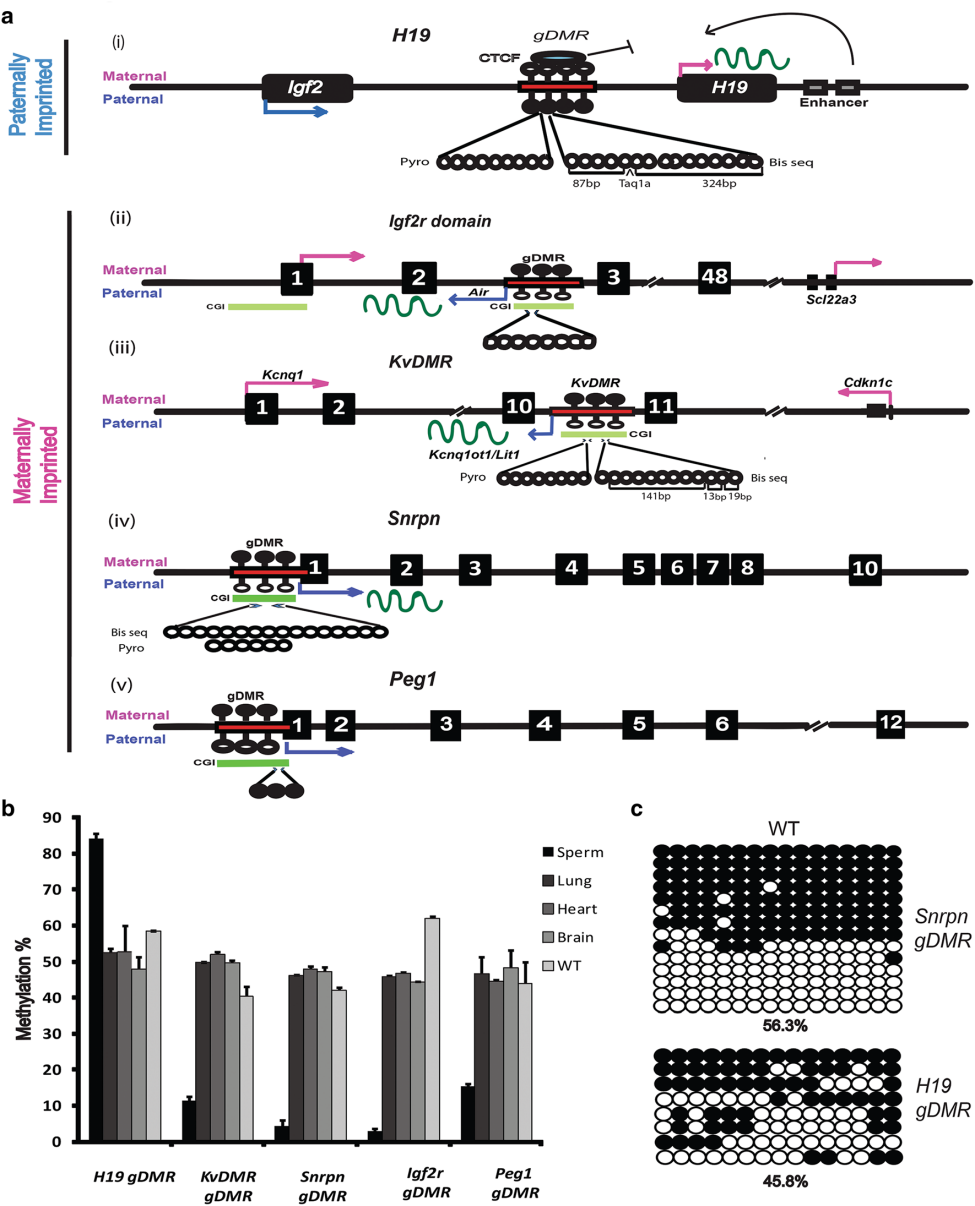
Five canonical imprinted regions in mouse and validation of gDMR methylation assays in WT ESCs. **a** Schematic showing the main features of the imprinted domains examined, along with positioning of imprinted gDMRs (*narrow rectangles*): (*i*) *H19/Igf2* region; (*ii*) *Igf2r* region; (*iii*) *KvDMR/Kcnq1* region; (*iv*) *Snrpn*; and (*v*) *Peg1*. Exons are represented by *dark-filled boxes*, and expression of the genes from maternal or paternal alleles is indicated by *bent arrows above* or *below* the *line*, respectively. *Wavy lines* and *narrow bars below* the *line* represent long non-coding RNA and CpG islands (CGIs), respectively. The number of CpG sites (*circles*) covered by pyrosequencing or clonal analysis, and enzyme restriction site positions and fragment sizes for COBRA are all shown below the gDMRs. *Filled circles* represent methylated sites, while *empty circles* are unmethylated. CCCTC-binding factor (CTCF) binds to *H19/Igf2* ICR on the maternal chromosome only (*i*) to block access of the downstream enhancers to *Igf2*. On the paternal chromosome, CTCF binding is blocked by methylation and the enhancers preferentially interact with *Igf2* rather than *H19*. **b** Methylation level of imprinted gDMRs in various mouse tissues and WT ESCs was quantified by pyrosequencing. Sperm was used as a control: all maternally imprinted genes exhibited low levels (<25%) of methylation and the paternally imprinted gene *H19* showed high levels (>75%) of methylation here, as expected. All maternally and paternally imprinted genes show ~50% methylation at gDMRs in lung, heart and brain as assessed by pyrosequencing. In WT ESC, methylation levels were very comparable, with only *Igf2r* a little high. *Error bars* indicate s.e.m. **c** Clonal analysis of *Snrpn* and *H19* gDMRs in parental WT ESCs. *Each circle* represents a CpG site assessed by bisulfite clonal analysis. Percentage total methylation is indicated at the *bottom of each panel*

We began our study by designing and validating pyrosequencing assays, as it is crucial that the designed primers cover the right regions at imprinted loci where methylation, once established, remains unchanged throughout development. To validate the approach, we initially chose five of the best-characterised imprinted loci for which extensive data on the gametic differentially methylated regions (gDMR) are available and which are representative of the different kinds of imprinted locus. The positioning of the gDMRs at these five imprinted loci is shown in Fig. 1. The paternally imprinted *H19* gDMR controls a small cluster of genes including *Igf2* [Fig. 1a(i)] and represents an insulator model of imprinting. On the maternal chromosome, CCCTC-binding protein (CTCF) binds to the gDMR, located intergenically, and forms an insulator to stop the interaction of the enhancer with the *Igf2* promoter. Such binding results in the silencing of *Igf2* on the maternal allele but allows the enhancers to activate *H19* (bent arrow) on the same allele. On the paternal chromosome, the ICR is methylated which prevents CTCF from binding; therefore, the enhancers can interact with *Igf2*, resulting in its transcription. The two parts of the intergenic gDMR covered by our pyrosequencing assay and by the clonal analysis/COBRA are also shown [Fig. 1a(i)]. Current indications are that many other imprinted genes seem likely to follow a non-coding RNA (ncRNA)-mediated model for regulation of imprinting. Two examples of this class are the maternally imprinted loci controlled by the *Igf2r* [Fig. 1a(ii)] [22] and *KvDMR* gDMRs [Fig. 1a(iii)], both located intragenically in introns. *Igf2r* and its neighbouring genes show maternal expression, and the *Igf2r* gDMR generates a paternally expressed non-coding transcript *Air* [Fig. 1a(ii)]. The full-length *Air* ncRNA and its transcription are required for the silencing of *Igf2r* and other neighbouring genes [29, 43]. *KvDMR* is the origin of a paternally expressed long ncRNA *Kcnq1ot1/Lit1* [Fig. 1a(iii)], which regulates imprinting at the *Kcnq1* locus. Truncation of *Kcnq1ot1* results in a loss of imprinting [44, 45]. Maternally imprinted *Snrpn* and *Peg1* represent another type of imprinted loci, where the gDMR is located directly at the promoter region of a gene, rather than intra- or intergenically. At these loci, methylation directly controls transcription [Fig. 1a(iv–v)].

### Methylation at imprinted gDMRs in WT ESCs is similar to that in normal tissues

Using pyrosequencing assays designed to match the known gDMR, we found that the *H19* gDMR (paternally imprinted) was hypermethylated (84.2%) in sperm samples, while all maternally imprinted gDMRs displayed very low methylation (Fig. 1b), as expected. All the gDMRs assayed also showed methylation around 50% (range normally observed 40–60% [46]) in somatic tissues and WT ESCs, although the level of *Igf2r* methylation was reproducibly higher in WT ESC. Further, we confirmed a normal level of methylation for *H19* and *Snrpn* gDMRs in WT ESCs by clonal analysis (Fig. 1c), which at 56.3 and 45.8%, respectively, was very comparable to that seen by pyrosequencing (58.5 and 42%) (Fig. 1b). These data: (1) indicated that the regions assayed by pyrosequencing showed the expected levels of methylation in somatic tissue, validating these assays, and (2) that the parental ESCs from which all the subsequent knockouts were derived had relatively normal levels of methylation at the gDMR.

### Comparable demethylation at imprinted loci in cells lacking DNMT3A/B or DNMT1

**Fig. 2:**
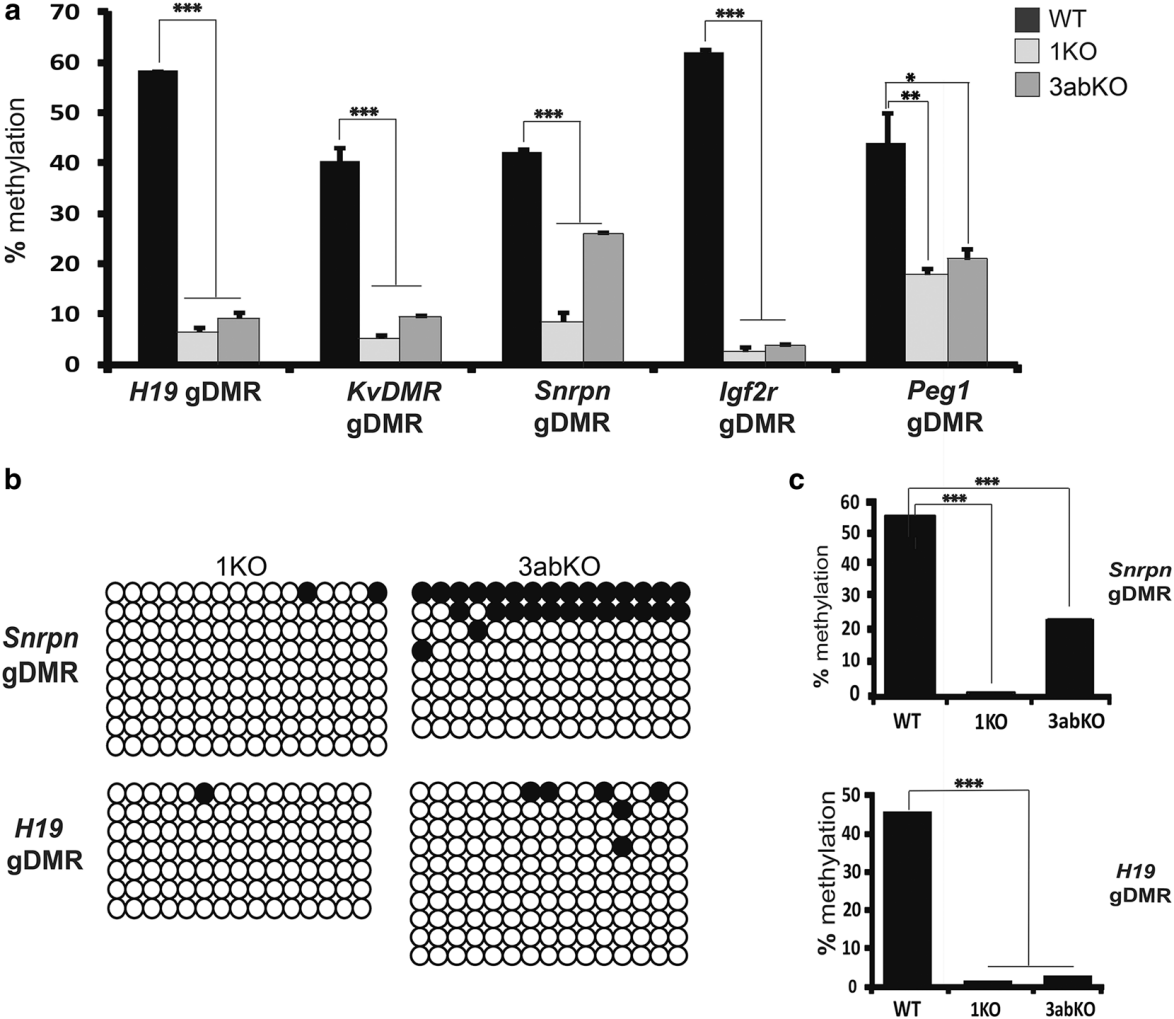
Mutation of *Dnmt3a/b* or *Dnmt1* gives comparable methylation loss at imprinted loci. **a** Pyrosequencing displaying methylation loss at various imprinted loci in DNMT1-deficient cells (1KO) and cells lacking both DNMT3A and 3B (3abKO). The methylation levels drop significantly for 1KO and 3abKO for all gDMRs assayed. **b** Clonal methylation analysis of imprinted gDMRs (*Snrpn* and *H19*) in 1KO and 3abKO ESCs. **c** Summary of methylation levels from the clonal data in **b**. There was a significant decrease in methylation for *H19* and *Snrpn* in all KO cell types compared to WT. *Error bars* indicate s.e.m. *p < 0.05; **p < 0.01; ***p < 0.001

Cells lacking DNMT1 (1KO) and as well as a recued cell line expressing a DNMT1 cDNA from an integrated transgene (1KO + 1) have been previously described [39]: the same authors describe cells lacking both DNMT3A and DNMT3B (3abKO) or rescued with DNMT3A2, and both protein levels and mRNA levels of the various proteins have been verified [39, 47]. Nevertheless, to ensure the cells have remained stable we verified the correct patterns of loss and rescue in the various cell lines using both westerns and RT-PCR (Additional file 3: Fig. S1). While previous studies have indicated a role for DNMT3A/B proteins in maintenance methylation at some repeats, and possibly at some other sequences in ESC, a potential maintenance role at imprinted gDMRs has not previously been examined in detail. All five gDMRs were found to be severely hypomethylated in 1KO and 3abKO cells. For the paternally imprinted *H19* gDMR (Fig. 2a), significant loss of methylation from 52.19% to less than 10% was observed for both cell types compared to WT ESCs (*p* value <0.05 for WT ESCs vs. 1KO and 3abKO). All maternal gDMRs also showed significant decreases in methylation in both KO cell types with *p* value <0.001 for WT ESCs versus either KO for all genes except *Peg1*, where *p* values were <0.01 and <0.05 for both 1KO and 3abKO respectively. At the *Igf2r* locus, methylation is almost completely lost in both types of knockout line compared to WT. We observed nevertheless a larger decrease in methylation for *Snrpn* in 1KO cells than 3abKO (8.7 vs. 26.1%) cells (Fig. 2b). To check this, we used clonal analysis and could confirm that the methylation level at *Snrpn* was lower in 1KO (1.4%) compared to 3abKO (24.16%) samples (Fig. 2c). The *H19* gDMR was equally hypomethylated in 1KO and 3abKO cells as shown by clonal analysis (*p* value <0.001 for WT versus 1KO or 3abKO) (Fig. 2c).

### Methylation can be restored following loss of DNMT3A/B, but not DNMT1

**Fig. 3:**
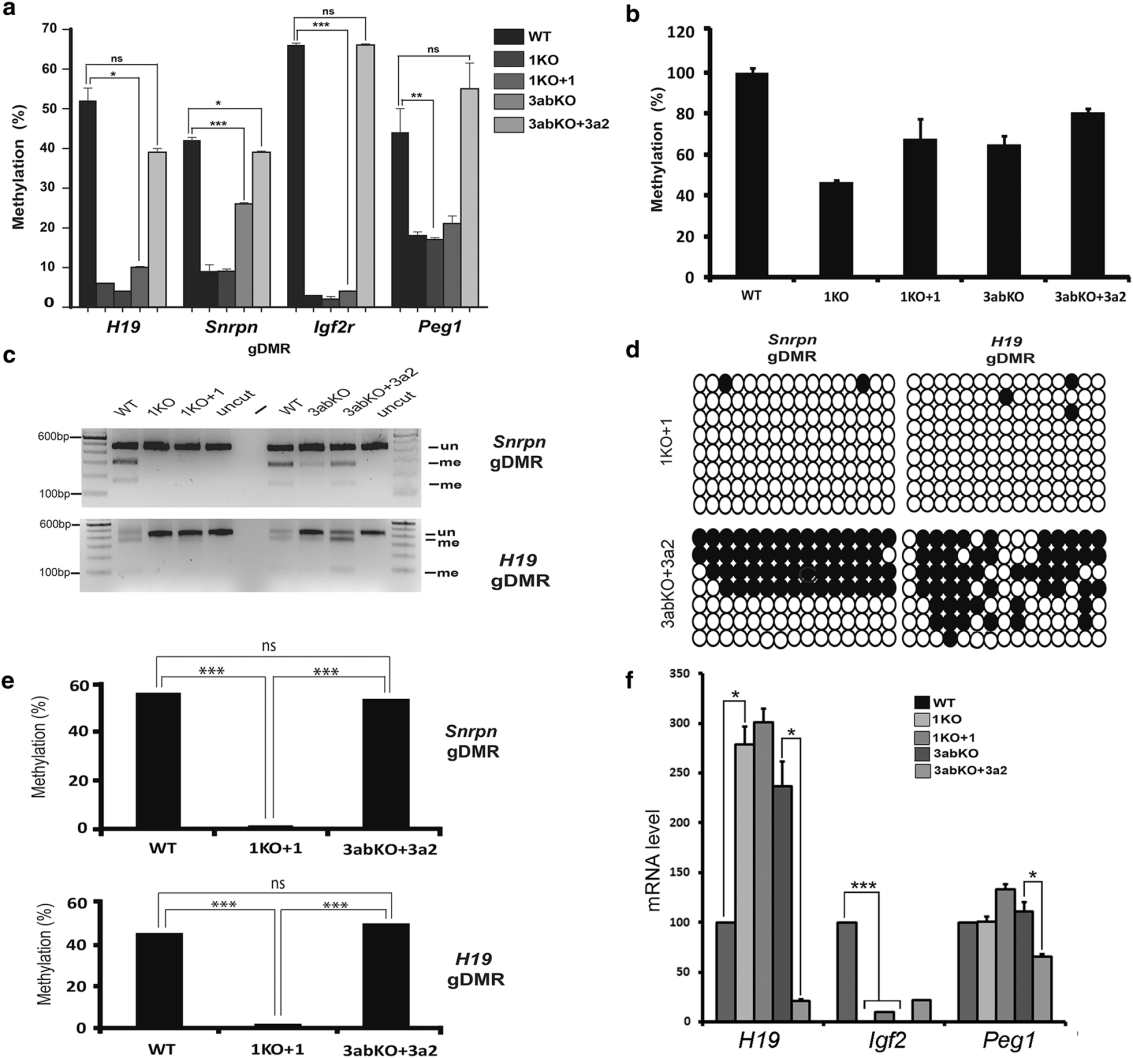
Despite similar levels of methylation, imprints can be restored only in 3abKO cells. **a** Methylation level assessed by pyrosequencing for parental (WT), DNMT1 rescued (1KO + 1) and 3abKO rescued (3abKO + 3a2) cells. Compared to WT, the methylation level remains significantly lower for all gDMRs in 1KO + 1 cells. All gDMRs show significant increase in methylation in 3abKO + 3a2 cells as compared to 1KO + 1. **b** Global DNA methylation in WT cells, 1KO, 3abKO and 3abKO + 3a2 rescued cells estimated by LUMA. **c** COBRA for two representative imprinted gDMRs, *H19* and *Snrpn.* Smaller fragments represent methylated DNA (me) and can be clearly seen in the WT and 3abKO + 3a2 lanes, but not in the 1KO + 1 lanes: un, unmethylated (D) clonal methylation analysis for *Snrpn* and *H19* showing methylation restoration in 3abKO + 3a2 cells and no recovery for 1KO + 1 cells. **e** Graphical summary of clonal data in **d**. **f** RT-qPCR for *H19*, *Igf2* and *Peg1* in the different ES lines. For *H19*, a significant increase in expression was observed in 1KO and 3abKO cells compared to WT (*p* value <0.05 for all). Rescue with *Dnmt3A2* (3abKO + 3a2), but not *Dnmt1* (1KO + 1), caused a significant decrease in transcription again (*p* value <0.05 3abKO + 3a2 vs. 3abKO cells). *Igf2* transcription is inversely linked to *H19* [Fig. 1a(i)] and as expected decreases significantly (*p* value <0.001 WT vs. 1KO and 3abKO) on loss of methylation in 1KO and 3abKO lines. Of the two rescue lines, 3abKO + 3a2 shows a greater recovery of transcription, though it fails to reach statistical significance. At the *Peg1* locus, there is an increase in transcription on loss of methylation (n.s.) and a significant decrease on reintroduction of DNMT3A2. *Error bars* indicate s.e.m in all panels: only significant changes are shown

As DNMT1 and DNMT3A/B appeared to have broadly similar roles in maintaining methylation marks at imprinted gDMRs in this system, we further aimed to investigate whether the loci responded in the same way to restoration of the respective enzymes. We first investigated the methylation levels of these gDMRs in 1KO ES cells and in those rescued with a cDNA expressing the full-length DNMT1 protein (1KO + 1) [48]. As previously reported by ourselves and others, imprinted gDMRs failed to restore methylation to normal WT levels (Fig. 3a). We also compared these results with 3abKO rescued cells (3abKO + 3a2) expressing the full-length DNMT3A2 protein [48]. Surprisingly, the maternally methylated *Igf2r* gDMR displayed complete recovery of methylation in 3abKO rescued cells, while for *Peg1*, gain of methylation in rescued cells (3abKO + 3a2) brought the levels to somewhat higher than WT level. There was in addition very substantial recovery of methylation at the paternally methylated *H19* gDMR (38.14%) as well as the *Snrpn* gDMR (38.88%) although recovery was not fully restored to normal WT levels (WT was 52.19% for *H19* and 42% *Snrpn*, respectively). The increase in methylation for all gDMRs in 3abKO + 3a2 cells was also very significant when compared to 1KO + 1 (Fig. 3a).

As methylation at gDMR was not completely restored to WT levels in the 3abKO + 3a2, we compared whole-genome methylation levels using LUMA [49], a bisulfite-independent quantitative assay that uses methylation-sensitive and methylation-insensitive enzymes to estimate total genomic methylation levels. LUMA analysis of 1KO and 3abKO cells shows clear loss of methylation compared to WT (set to 100%). While both 1KO + 1 and 3abKO + 3a2 cells showed gain in methylation, neither fully recovered to WT levels (Fig. 3b). HCT116 WT and DKO were used to confirm the LUMA was working: as expected, HCT116WT was found to be hypermethylated and methylation drops significantly for DKO cells lacking DNMT1 and DNMT3B (data not shown). To further confirm our results with respect to the imprinted gDMR, we carried out COBRA on *Snrpn* and *H19* gDMRs, which indicated clear losses of methylation in both KO cell types, no recovery in 1KO + 1 and almost complete methylation restoration in 3abKO + 3a2 samples (Fig. 3c). We also used clonal analysis for these gDMRs, which gave similar results (Fig. 3d, e).

### Transcriptional activity tracks with methylation at the *H19* locus

To test whether the loss of these methyltransferases and their recovery are associated with abnormal expression, we carried out RT-qPCR on a number of imprinted loci. While most imprinted genes tested were not transcribed at significant levels in these ESC, precluding assessment of response, we did find that the expression level of *H19* was significantly higher in 1KO and 3abKO cells as compared to WT, consistent with biallelic expression of *H19* in those cell lines (Fig. 3f). Rescuing 1KO cells with DNMT1 did not restore repression but rescuing 3abKO cells with DNMT3A significantly reduced (*p* < 0.05) levels of *H19* mRNA (Fig. 3f). In keeping with the regulatory mechanism in place at this locus [Fig. 1a(i)], *Igf2* showed the opposite pattern, with repression in 1KO and 3abKO cells and greatest recovery in 3abKO + 3a2 cells (Fig. 3f), though this did not reach significance because of the lower transcription levels. In addition, the *Peg1* mRNA, which is mildly depressed in the 3abKO line, showed a significant repressive effect of adding back in the DNMT3A2 enzyme (Fig. 3f).

### Failure to restore methylation at *KvDMR*

**Fig. 4:**
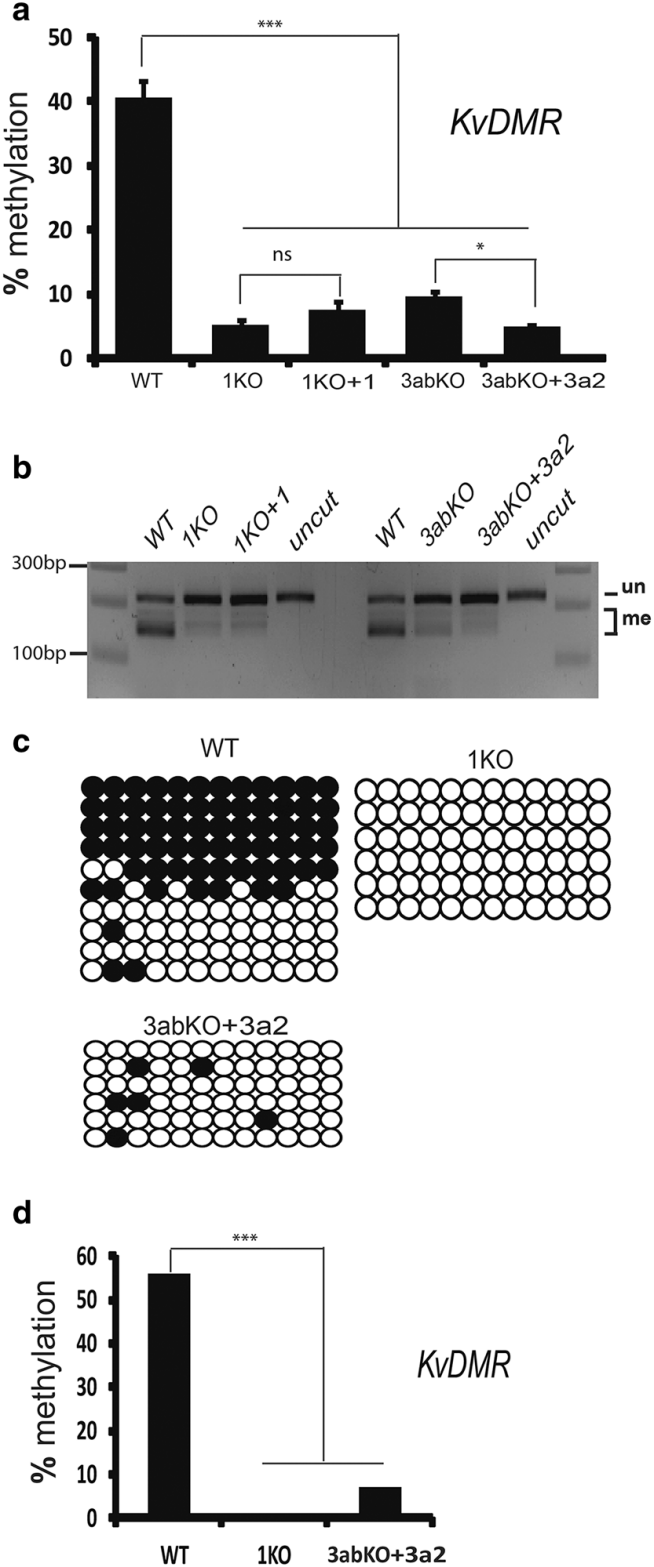
Rescued 3abKO cells are inefficient at re-establishing imprints on the KvDMR gDMR suggesting a unique regulatory mechanism associated with this imprinted locus. **a** Methylation level of *KvDMR* in various ES cell lines by pyrosequencing. The gDMR exhibits significant loss of methylation in 1KO and 3abKO cells, and methylation remains unchanged in both rescued lines. **b** Validation of pyrosequencing results by COBRA; *Taq*a*I* was used to digest the PCR product: me, fragments resulting from cleavage of methylated product; un, fragments when unmethylated. There is no increase in methylated fragments from 3abKO to 3abKO + 3a2 samples. **c** Clonal analysis for *KvDMR* in WT, 1KO and 3abKO + 3a2 cells. **d** Graphical summary of clonal data from **c**. *Error bars* indicate s.e.m

The *KvDMR* gDMR showed a normal level of methylation in WT ESCs, loses methylation in 1KO (5.3%) and does not regain methylation in 1KO + 1 cells (Fig. 4a) as for the other imprinted loci. While this gDMR also showed a very low level of methylation in 3abKO (9.5%), unlike the other four benchmark loci examined, rescue with DNMT3A2 in 3abKO cells failed to reinstate methylation at this maternally imprinted locus (Fig. 4a). Differences in methylation were significant for WT versus 1KO, 1KO + 1, 3abKO and 3abKO + 3a2 (*p* value <0.001) and non-significant for 1KO versus 1KO + 1, 1KO versus 3abKO and 1KO + 1 versus 3abKO + 3a2 (Fig. 4a). A small but significant decrease in methylation was observed in 3abKO + 3a2 cells compared to 3abKO cells, with a *p* value <0.05. We further verified these results overall using COBRA; a clear loss of methylation was observed in 1KO and 3abKO cells with no methylation restoration for either rescued cell type (Fig. 4b). *KvDMR* was also found via clonal analysis to be 56, 0 and 7% methylated in WT, 1KO + 1 and 3abKO + 3a2 cells, respectively (Fig. 4c), with differences in methylation between WT and rescued cell lines remaining very significant (*p* value <0.001) (Fig. 4c, d). These results suggest that a unique mechanism may be associated with this maternally imprinted locus.

### Loss of methylation of remaining known and putative gDMR in Dnmt3ab KO cells

**Fig. 5:**
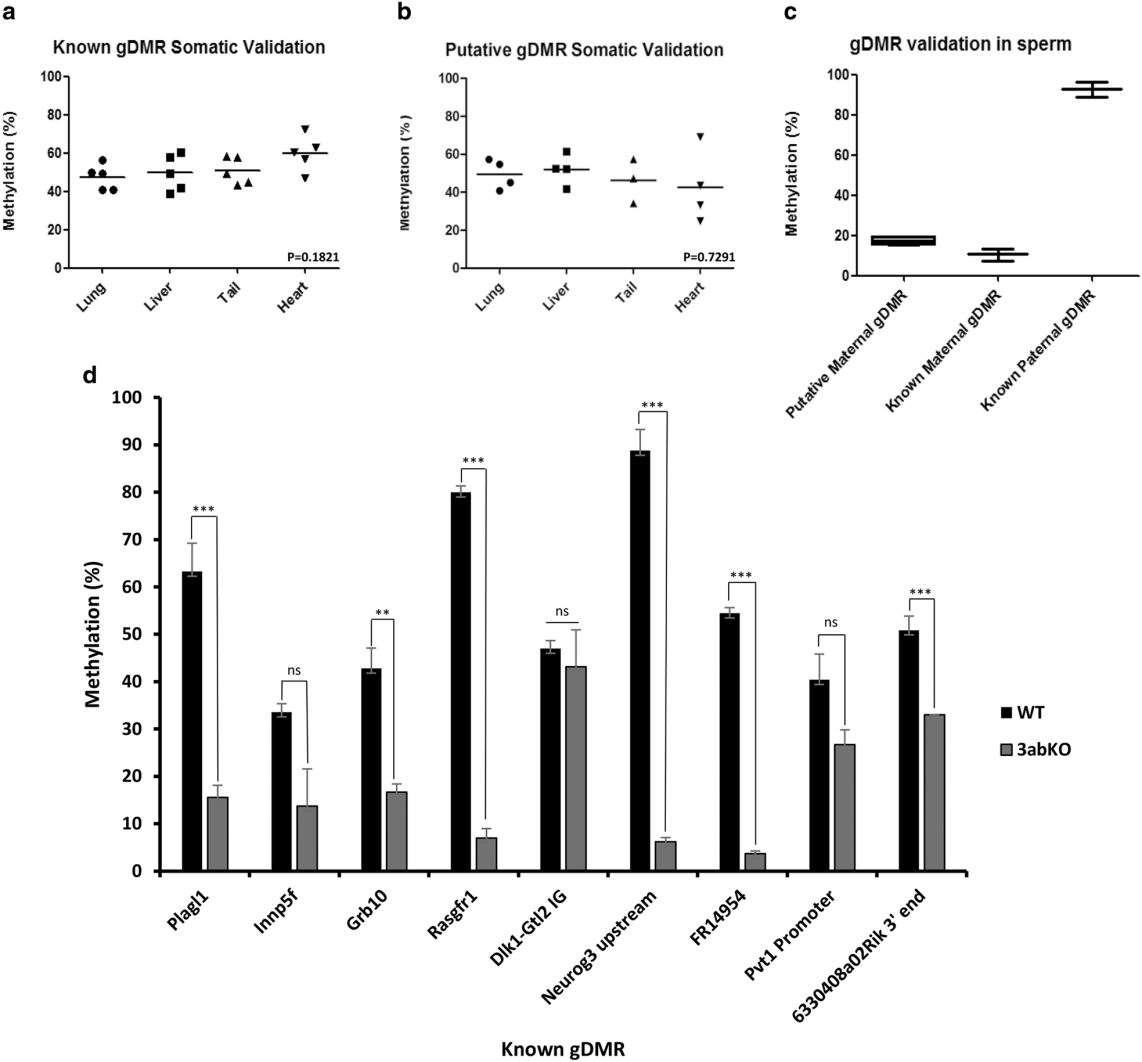
*Examination by pyroassay of remaining known and putative imprinted gDMR*. **a** Validation of pyrosequencing assays for remaining known imprinted gDMRs. Average methylation levels across all CpG in each assay were plotted. The median for all assays is indicated by a *horizontal line*, and these did not significantly differ from one another using Kruskal–Wallis (*p* value 0.1821). **b** Results for putative imprinted gDMR, plotted as in **a**. While differences between medians are not significant (Kruskal–Wallis *p* value 0.7291), a greater variance can be seen, particularly in heart. **c** Verification that pyrosequencing assays for both known and putative maternal gDMR showed low methylation in sperm, while the paternal assay returned high methylation levels. **d** Methylation is lost at all known and putative gDMR, though the decrease is very small at *Dlk1*-*Gtl2 IG*. *Error bars* represent s.e.m.; **p < 0.01; ***p < 0.001; *n.s.* not significant

To determine whether recovery of methylation in 3abKO + 3a2 rescue cells is a general phenomenon for imprinted loci, we wished to extend our study to include all known imprints as well as the putative imprinted loci recently identified by Wang et al. [15]. To this end, we designed pyrosequencing assays for all the remaining known and putative gDMR based on the coordinates indicated in the latter. Assays were excluded which (1) gave low scores in the design software and poor peaks on assaying; (2) displayed methylation values outside ±1.5 times the SD from the 50% methylation expected in a range of mouse somatic tissues [46]; and (3) did not show >75% (paternal) or <25% (maternal) methylation in sperm samples. The results for the remaining five known loci we could assay in somatic tissues (*Plagl1*, *Innp5f*, *Grb10*, *Rasgrf1* and *Dlk1*-*Gtl2 IG*) are shown in Fig. 5a and displayed relatively tight clustering around the median, which was noticeably higher in heart tissue.

Assays designed to cover four novel putative gDMRs described in Wang et al. [15] (*Neurog3* upstream, *FR149454* promoter, *Pvt1* promoter and *6330408a02Rik* 3′ end) also validated in a range of mouse somatic tissue (Fig. 5b), with average values for the assays falling within the threshold criteria as per Fig. 5a. Notably, there was greater variation in the methylation values for these loci than for the known gDMR. There was less deviation from the median methylation value in liver than heart tissue, which has consistently exhibited a wider spread of results (Fig. 5a, b). The results for the known and putative imprints in mouse sperm samples are shown in Fig. 5c, where they displayed low (22.8% or below) methylation for maternally methylated gDMRs, or conversely hypermethylation (78%) at the respective paternally methylated regions.

With our validated assays, we next examined these nine additional known or putative gDMRs in the mouse ESCs. All of these loci showed decreases in methylation in 3abKO cells when compared to WT (Fig. 5d), though differences at some (*Innp5f*, *Dlk1*-*Gtl2 IG* and *Pvt1* promoter) did not reach statistical significance.

### Methylation can be restored following loss of DNMT3A/B at the majority of imprinted gDMR in DNMT3A2-rescued ES cells

**Fig. 6:**
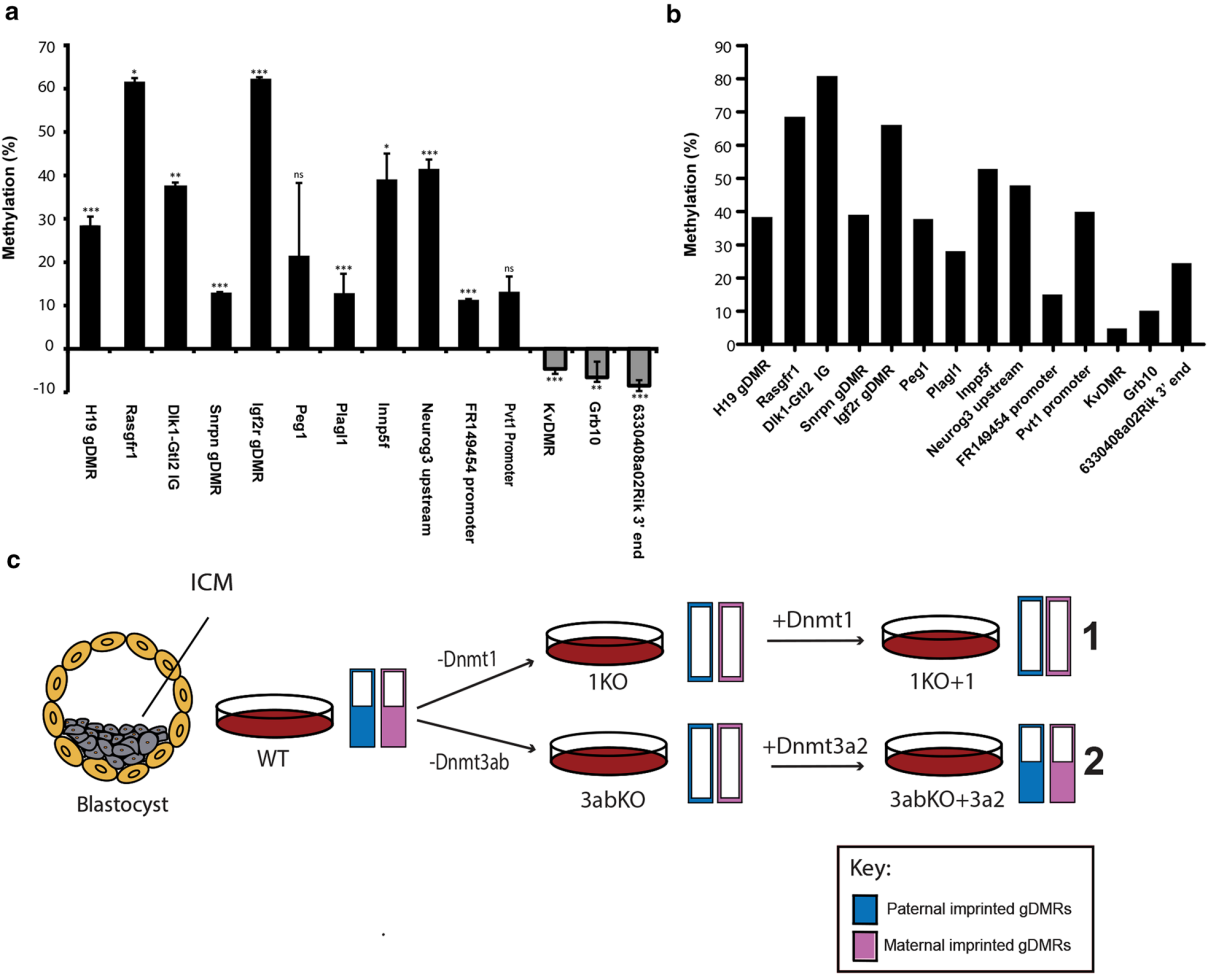
Summary of methylation responses at known and putative imprinted gDMR. **a** Changes in methylation seen in 3abKO cells rescued with DNMT3A2 for all of the known and putative gDMRs. Eleven gDMRs of fourteen which could be assayed showed gains in methylation, with nine of these reaching significance. *KvDMR, Grb10* and *6330408a02Rik* failed to recover methylation levels, instead showing significant additional reductions in methylation when compared to the 3abKO ES cells. **b** Absolute methylation levels in the 3abKO + 3a2 cells at the various gDMR. **c** Schematic summarising the changes in methylation seen in the two types of knockout and rescue. WT ESC cells grown in petri dishes were derived originally from inner cell mass (ICM) of early embryo and retained 50% methylation at most imprinted gDMR (*half-filled bars*: paternal at *left*, maternal at *right*). While loss of DNMT1 (pathway 1, *top*) gave comparable hypomethylation to loss of DNMT3A/B (pathway 2, *bottom*), no recovery of methylation at either paternally or maternally methylated imprinted gDMRs was seen in DNMT1-rescued cells, whereas rescue with DNMT3A2 in 3abKO cells restored methylation non-discriminately at both paternally and maternally imprinted gDMRs (pathway 2)

Our initial work reported above established a clear ability for DNMT3A2 to restore methylation marks at key imprinted gDMRs such as *Igf2r* in 3abKO cells. We now extended this analysis to the other known and putative gDMRs indicated above. For convenience, all of the assays from our work are presented together in Fig. 6a. Eight of 10 known gDMRs assayed gained methylation when compared to 3abKO (Fig. 6a), with only *Grb10*, in addition to *KvDMR*, showing a failure to regain methylation. Of the four putative gDMR assayed, only one (*6330408a02Rik*) did not show any increase in methylation. The maternal gDMR *Igf2r* showed the largest recovery of methylation when compared to the 3abKO ES cells at 62.30%. Gain in methylation was seen at all the known paternally methylated gDMRs assayed-*H19*, *Rasgrf1* and *Dlk1*-*Gtl2 IG* (Fig. 6a). Absolute methylation values for the 3abKO + 3a2 cells are shown in Fig. 6b for comparison. Notably, *KvDMR, Grb10* and *6330408a02Rik 3′* end not only fail to regain methylation (Fig. 6a), but instead continue to lose it in the 3abKO + 3a2 cell. This suggests that in the absence of DNMT3A/B the loci do not remain stable but rather continue to lose methylation (Fig. 6a). Our findings for the individual loci are summarised in Fig. 6c and in Table 1.

**Table 1:** Summary of findings with regard to methylation at gametic differentially methylated regions at imprinted loci

Locus	Chr	Chromosomal region^a^ delineated by pyro assay	Origin^b^	gDMR status^c^	Sperm meth %	Somatic meth %^d^	Location within gene	CpG island	Gain in 3abKO + 3a2 ESCs
*H19*	7	142,580,262–142,580,434	P	Known	84.19	51.06	Intergenic	No	Yes
*Rasgrf1*	9	89,872,365–89,872,512	P	Known	89.00	52.67	Intergenic	No	Yes
*Dlk1*-*Gtl2* IG	12	109,528,521–109,528,661	P	Known	96.33	57.92	Intergenic	Yes	Yes
*Snrpn*	7	60,004,993–60,005,163	M	Known	4.41	43.42	Promoter/Exon 1	Yes	Yes
*Igf2r*	17	12,960,690–12,962,806	M	Known	3.00	45.71	Intronic	Yes	Yes
*Peg1*	6	30,687,444–30,688,524	M	Known	15.50	51.75	Intronic	Yes	Yes
*Plagl1*	10	13,091,014–13,091,154	M	Known	10.80	42.83	Promoter/Exon 1	Yes	Yes
*Inpp5f*	7	128,688,173–128,688,290	M	Known	13.10	57.19	Intronic	Yes	Yes
*Grb10*	11	12,025,894–12,026,044	M	Known	7.38	45.80	Intronic	Yes	No
*KvDMR*	7	143,295,771–143,295,910	M	Known	6.33	50.54	Intronic	Yes	No
*6330408a02Rik* 3′	7	13,260,963–13,261,135	M	Putative	19.43	47.71	Exon 13	Yes	No
*Neurog3* upstream	10	62,127,922–62,128,093	M	Putative	15.44	43.84	Intragenic	No	Yes
*FR149454* promoter	11	119,258,958–119,259,182	M	Putative	15.33	58.67	Intronic	No	Yes
*Pvt1* promoter	15	62,037,136–62,037,311	M	Putative	19.42	43.30	Intragenic	No	Yes

Data are presented for each gDMR for which a validated pyrosequencing assay (pyro assay) could be established. Known gDMR are listed first and then putative, with paternal imprints preceding maternal (none of the putative gDMRs were paternal)

*Chr* chromosome, *Origin* parent of origin of methylation mark, *meth* methylation

^a^mm10 release

^b^Parental origin of methylation: *p* paternal chromosome, *m* maternal

^c^Whether gDMR is well characterised (known) or recently discovered (putative)

^d^Somatic methylation = average methylation value across three adult tissues

## Discussion

Maintenance methylation is a vital process as it is responsible for the stable inheritance of this epigenetic signature from mother to daughter cells during the process of mitosis. At one time, DNMT1 was thought to be the only enzyme associated with maintenance of methylation due to its preferential binding to hemi-methylated DNA and its presence at the replication foci [4, 5]. Chen et al. [39] showed, however, that DNMT3A and DNMT3B were also important for maintenance methylation at some repeats and, using a qualitative technique, at certain imprinted loci. In a previous study, we confirmed that DNMT3A and 3B were needed at a few selected imprinted loci using a more quantitative approach and extended this observation to transiently imprinted genes, which also require DNMT3A/B for maintenance in ESCs [13]. Here we looked in greater depth at all the known gametic DMR as well as some newly identified imprinted gDMR and confirm their reliance (with 1–2 exceptions such as *Dlk1*-*Gtl2* IG) on the DNMT3A/B enzymes for maintenance of methylation. Interestingly, the decrease in methylation at these gDMRs was found to be approximately the same in 1KO and 3abKO cells, suggesting an equal contribution by DNMT3A/B and DNMT1 in maintenance of methylation at imprinted gDMRs in ESCs.

Overexpression of DNMT1 in 1KO cells resulted in a global increase in methylation as reported previously; similar global increases in methylation were observed here in DNMT3A/B rescued cells using LUMA, although this increase does not bring the methylation level to the normal WT level globally. This could be due to a number of reasons: (1) it may indicate the presence of some sequences which are refractory to remethylation in ESCs; (2) the expression of the cDNA in the rescued cells may not be as high as the endogenous levels of DNMT3A2; and/or (3) some sequences may require both DNMT3B and DNMT3A2 to fully recover methylation to WT levels [11].

We showed here that in the 1KO + 1 cells there was no gain in methylation seen at any of the gDMR examined, confirming earlier results from a number of groups. In contrast, 3abKO cells rescued with DNMT3A2 showed clear and reproducible gains in methylation at the majority of imprinted gDMR. These results were confirmed using up to three techniques per locus-pyrosequencing, clonal analysis and COBRA. Additionally, the transcriptional status of *H19* and *Igf2* responded appropriately to the loss and regain of methylation, confirming that functional imprinting was being affected, at least at these loci (other loci showed transcription levels which were too low to reliably quantitate in these cells). While some previous studies have found that none [38] or only one [39] imprinted locus showed any gains in methylation on rescue, these were based on more qualitative techniques and in many cases could not examine the locus except at a low level of resolution using techniques such as Southern blotting. Here we show gain in methylation of greater than 10% at 11/14 gDMR, with substantially greater gains at most. Average gain was 28%, which in the context of an incomplete overall rescue as indicated from the global methylation levels (above) represents a corrected gain of close to 50%.

There were a few loci (3/14-*KvDMR*, *Grb10 *and *6330408a02Rik *3′ end), which showed no gain in methylation, and in fact displayed evidence for further hypomethylation relative to the 3abKO cells. This latter is not unexpected since we have shown that ESC rely on DNMT3A/B for maintenance methylation as well as de novo activity, so if these three loci are refractory to the action of DNMT3A2 in the rescued line, they would be expected to continue to lose methylation. Our examination of ENCODE data and of the current literature has found so far no common denominator for these three loci. Nevertheless, these results show that for the majority of imprinted loci, methylation at the gDMR, and in some cases functional imprinting, can be restored in a somatic cell type without germline passage.

What mechanism is associated with imprint recovery in 3abKO + 3a2 and not 1KO + 1 cells? This is particularly puzzling since the two rescued cell types both have all three enzymes present. Two possibilities are that (1) loss of DNMT3A/B proteins could alter histone marks on chromatin, which then act to attract de novo methylation by DNMT3A2 on rescue or (2) loss of DNMT1 protein causes a change in histone marks, which mean that even after rescue, the DNA cannot be remethylated. Notably, triple KO cell lines lacking all three enzymes also fail to show imprint restoration when rescued [39], suggesting that it is the loss of DNMT1 which leads to an irreversible change in epigenetic potential, precluding rescue with DNMT3A2. It has been reported that the loss of DNMT1 results in loss of H3K9me3 in ESC [50]. One possibility is that loss of H3K9me3 occurs in 1KO cells, but not 3abKO cells, and that the presence of this mark facilitates remethylation by DNMT3A2 in the latter. It has also been reported that the PWWP domain of DNMT3A is linked with targeting of chromatin carrying H3K36me3 [51]. Loss and gain of methylation marks on imprinted gDMRs could be due to the presence and absence of such interactions between methyltransferases and histone marks associated with chromatin, which require further experimental exploration in this system.

We clearly identified three gDMR, including *KvDMR*, where methylation once lost cannot be recovered. This supports other evidence, suggesting that mechanism of imprinting and response to methylation loss and recovery can vary among imprinted genes [52]. In future, it will be interesting to compare the histone marks associated with *KvDMR* and with those associated with gDMRs that recover methylation in rescued cells. The *Dlk1*-*Gtl2* IG was also interesting in that it showed overmethylation in our experiments, gaining almost 40% methylation in 3abKO + 3a2. The tendency of this locus to become hypermethylated in human ES and iPS cells has been noted before [53, 54] and may reflect some fundamental mechanistic feature of imprinting at this locus, which in practise could act as a barrier to somatic reprogramming efforts.

During the course of writing, a paper from the Wong group investigating the behaviour of UHRF1 rescue cells found that a number of imprinted genes showed gains in methylation in that system too [55]. Methylation gain was only seen at some of the imprinted loci, and there was no clear link to the location of the gDMR, the presence of antisense transcripts or the type of imprint. Furthermore, they investigated common histone marks and found no relationship between any specific mark and the ability of the locus to gain methylation in the rescues. They did not, however, investigate transcriptional changes at the loci in their cells. Their data, taken together with the findings we present here, show that gametically acquired methylation at imprinted loci can be reset somatically in certain circumstances.

## Conclusions

We have shown that (1) both DNMT1 and DNMT3A/B loss generate similar methylation changes at imprinted gDMRs in ESCs; (2) recovery of imprints in 1KO + 1 cell lines is not possible but imprints can be recovered in DNMT3A2-rescued 3abKO cells; and (3) there are some exceptional gDMRs where imprints, once lost, cannot be re-established. Our findings highlight important differences between the two cell systems and indicate that it may be possible to restore imprints somatically under certain circumstances, an observation of clear relevance for imprinting disorders. This may provide a useful model system in which to further explore reprogramming.

## Additional files

**Additional file 1: Table S1.** Details of the primers used for RT-qPCR during the study.

**Additional file 2: Table S2.** Details of the primers used for pyrosequencing during the study. The gametic differentially methylated region (gDMR) is given at left. Some primer sets are commercially available from Qiagen, as indicated. The sequence of the unconverted DNA, as well as the bisulfite-converted sequence, is given for ease of identification. Which primer carried the biotin modification is also indicated.

**Additional file 3: Figure S1.** Controls to confirm the methyltransferase activities in the ESC used during the study. (A) Westerns showing the presence or absence of DNMT1 (top) or of DNMT3A2 (bottom) in the various cell lines indicated. ACTB and GAPDH are loading controls. The size of the expected protein is shown at left. A number of DNMT3B antibodies tried proved unreliable. (B) RT-PCR showing the presence or absence of transcripts for the various enzymes in the ESC used. The expected sizes are shown at left. *Hprt* was a loading control.

## References

[1] Smith ZD, Meissner A (2013). DNA methylation: roles in mammalian development. Nat Rev.

[2] Yoder JA, Bestor TH (1998). A candidate mammalian DNA methyltransferase related to pmt1p of fission yeast. Hum Mol Genet.

[3] Bestor TH (2000). The DNA methyltransferases of mammals. Hum Mol Genet.

[4] Leonhardt H, Page AW, Weier HU, Bestor TH (1992). A targeting sequence directs DNA methyltransferase to sites of DNA replication in mammalian nuclei. Cell.

[5] Lei H, Oh SP, Okano M, Juttermann R, Goss KA, Jaenisch R (1996). De novo DNA cytosine methyltransferase activities in mouse embryonic stem cells. Development.

[6] Hermann A, Goyal R, Jeltsch A (2004). The Dnmt1 DNA-(cytosine-C5)-methyltransferase methylates DNA processively with high preference for hemimethylated target sites. J Biol Chem.

[7] Okano M, Xie S, Li E (1998). Cloning and characterization of a family of novel mammalian DNA (cytosine-5) methyltransferases. Nat Genet.

[8] Chen T, Ueda Y, Xie S, Li E (2002). A novel Dnmt3a isoform produced from an alternative promoter localizes to euchromatin and its expression correlates with active de novo methylation. J Biol Chem.

[9] Robert M-F, Morin S, Beaulieu N, Gauthier F, Chute IC, Barsalou A (2003). DNMT1 is required to maintain CpG methylation and aberrant gene silencing in human cancer cells. Nat Genet.

[10] Kaneda M, Okano M, Hata K, Sado T, Tsujimoto N, Li E (2004). Essential role for de novo DNA methyltransferase Dnmt3a in paternal and maternal imprinting. Nature.

[11] Borgel J, Guibert S, Li Y, Chiba H, Schübeler D, Sasaki H, et al. Targets and dynamics of promoter DNA methylation during early mouse development. Nat. Genet. 2010;42:1093–100. http://www.nature.com/doifinder/10.1038/ng.708. 10.1038/ng.708 21057502

[12] Chen T, Ueda Y, Dodge JE, Wang Z, Li E. Establishment and maintenance of genomic methylation patterns in mouse embryonic stem cells by Dnmt3a and establishment and maintenance of genomic methylation patterns in mouse embryonic stem cells by Dnmt3a and Dnmt3b. 2003. 10.1128/MCB.23.16.5594-5605.2003 PMC166327 12897133

[13] Rutledge CE, Thakur A, O’Neill KM, Irwin RE, Sato S, Hata K (2014). Ontogeny, conservation and functional significance of maternally inherited DNA methylation at two classes of non-imprinted genes. Development.

[14] Olek A, Walter J (1997). The pre-implantation ontogeny of the H19 methylation imprint. Nat Genet.

[15] Wang L, Zhang J, Duan J, Gao X, Zhu W, Lu X (2014). Programming and inheritance of parental DNA methylomes in mammals. Cell.

[16] Shemer R, Birger Y, Riggs AD, Razin A (1997). Structure of the imprinted mouse Snrpn gene and establishment of its parental-specific methylation pattern. Proc Natl Acad Sci USA.

[17] Guo F, Li X, Liang D, Li T, Zhu P, Guo H, et al. Active and passive demethylation of male and female pronuclear DNA in the mammalian zygote. Cell Stem Cell. 2014;15:447–58. http://www.sciencedirect.com/science/article/pii/S1934590914003415. 10.1016/j.stem.2014.08.003 25220291

[18] Smith ZD, Chan MM, Mikkelsen TS, Gu H, Gnirke A, Regev A (2012). A unique regulatory phase of DNA methylation in the early mammalian embryo. Nature.

[19] Okano M, Bell DW, Haber DA, Li E (1999). DNA methyltransferases Dnmt3a and Dnmt3b are essential for de novo methylation and mammalian development. Cell.

[20] Stewart CL, Stuhlmann H, Jähner D, Jaenisch R (1982). De novo methylation, expression, and infectivity of retroviral genomes introduced into embryonal carcinoma cells. Proc Natl Acad Sci USA.

[21] Santos F, Hendrich B, Reik W, Dean W (2002). Dynamic reprogramming of DNA methylation in the early mouse embryo. Dev Biol.

[22] Bartolomei MS, Ferguson-Smith AC (2011). Mammalian genomic imprinting. Cold Spring Harb Perspect Biol.

[23] John RM, Lefebvre L (2011). Developmental regulation of somatic imprints. Differentiation.

[24] Davis TL, Yang GJ, McCarrey JR, Bartolomei MS (2000). The H19 methylation imprint is erased and re-established differentially on the parental alleles during male germ cell development. Hum Mol Genet.

[25] Adalsteinsson B, Ferguson-Smith A (2014). Epigenetic control of the genome—lessons from genomic imprinting. Genes.

[26] Smallwood SA, Kelsey G (2012). De novo DNA methylation: a germ cell perspective. Trends Genet.

[27] Sutcliffe JS, Nakao M, Christian S, Orstavik KH, Tommerup N, Ledbetter DH (1994). Deletions of a differentially methylated CpG island at the SNRPN gene define a putative imprinting control region. Nat Genet.

[28] Buiting K, Saitoh S, Gross S, Dittrich B, Schwartz S, Nicholls RD (1995). Inherited microdeletions in the Angelman and Prader–Willi syndromes define an imprinting centre on human chromosome 15. Nat Genet.

[29] Zwart R, Sleutels F, Wutz A, Schinkel AH, Barlow DP (2001). Bidirectional action of the Igf2r imprint control element on upstream and downstream imprinted genes. Genes Dev.

[30] Bartolomei MS (2009). Genomic imprinting: employing and avoiding epigenetic processes. Genes Dev.

[31] Forne T, Oswald J, Dean W, Saam JR, Bailleul B, Dandolo L (1997). Loss of the maternal H19 gene induces changes in Igf2 methylation in both cis and trans. Proc Natl Acad Sci USA.

[32] Bourc’his D, Xu GL, Lin CS, Bollman B, Bestor TH (2001). Dnmt3L and the establishment of maternal genomic imprints. Science.

[33] Hata K, Okano M, Lei H, Li E (2002). Dnmt3L cooperates with the Dnmt3 family of de novo DNA methyltransferases to establish maternal imprints in mice. Development.

[34] Kaneda M, Okano M, Hata K, Sado T, Tsujimoto N, Li E (2004). Essential role for de novo DNA methyltransferase Dnmt3a in paternal and maternal imprinting. Nature.

[35] Kato Y, Kaneda M, Hata K, Kumaki K, Hisano M, Kohara Y (2007). Role of the Dnmt3 family in de novo methylation of imprinted and repetitive sequences during male germ cell development in the mouse. Hum Mol Genet.

[36] Smallwood SA, Tomizawa S, Krueger F, Ruf N, Carli N, Segonds-Pichon A (2011). Dynamic CpG island methylation landscape in oocytes and preimplantation embryos. Nat Genet.

[37] Wernig M, Meissner A, Foreman R, Brambrink T, Ku M, Hochedlinger K (2007). In vitro reprogramming of fibroblasts into a pluripotent ES-cell-like state. Nature.

[38] Tucker KL, Beard C, Dausmann J, Jackson-Grusby L, Laird PW, Lei H (1996). Germ-line passage is required for establishment of methylation and expression patterns of imprinted but not of nonimprinted genes. Genes Dev.

[39] Chen T, Ueda Y, Dodge JE, Wang Z, Li E (2003). Establishment and maintenance of genomic methylation patterns in mouse embryonic stem cells by Dnmt3a and Dnmt3b. Mol Cell Biol.

[40] Bustin SA (2000). Absolute quantification of mRNA using real-time reverse transcription polymerase chain reaction assays. J Mol Endocrinol.

[41] Walsh CP, Bestor TH. Cytosine methylation and mammalian development. Genes Dev. 1999;13:26–34. http://www.genesdev.org/cgi/content/full/13/1/26. 10.1101/gad.13.1.26 PMC316374 9887097

[42] Giardine B, Riemer C, Hardison RC, Burhans R, Elnitski L, Shah P (2005). Galaxy: a platform for interactive large-scale genome analysis. Genome Res.

[43] Sleutels F, Zwart R, Barlow DP (2002). The non-coding Air RNA is required for silencing autosomal imprinted genes. Nature.

[44] Mancini-Dinardo D, Steele SJS, Levorse JM, Ingram RS, Tilghman SM (2006). Elongation of the Kcnq1ot1 transcript is required for genomic imprinting of neighboring genes. Genes Dev.

[45] Shin J-Y, Fitzpatrick GV, Higgins MJ (2008). Two distinct mechanisms of silencing by the KvDMR1 imprinting control region. EMBO J.

[46] Woodfine K, Huddleston JE, Murrell A (2011). Quantitative analysis of DNA methylation at all human imprinted regions reveals preservation of epigenetic stability in adult somatic tissue. Epigenetics Chromatin.

[47] Tsumura A, Hayakawa T, Kumaki Y, Takebayashi S, Sakaue M, Matsuoka C (2006). Maintenance of self-renewal ability of mouse embryonic stem cells in the absence of DNA methyltransferases Dnmt1, Dnmt3a and Dnmt3b. Genes Cells.

[48] Oda M, Yamagiwa A, Yamamoto S, Nakayama T, Tsumura A, Sasaki H (2006). DNA methylation regulates long-range gene silencing of an X-linked homeobox gene cluster in a lineage-specific manner. Genes Dev.

[49] Karimi M, Luttropp K, Ekström TJ (2011). Global DNA methylation analysis using the luminometric methylation assay. Methods Mol Biol.

[50] Quenneville S, Verde G, Corsinotti A, Kapopoulou A, Jakobsson J, Offner S (2011). In embryonic stem cells, ZFP57/KAP1 recognize a methylated hexanucleotide to affect chromatin and DNA methylation of imprinting control regions. Mol Cell.

[51] Ge YZ, Pu MT, Gowher H, Wu HP, Ding JP, Jeltsch A (2004). Chromatin targeting of de novo DNA methyltransferases by the PWWP domain. J Biol Chem.

[52] Bartolomei MS, Ferguson-Smith AC. Mammalian genomic imprinting. Cold Spring Harb Perspect Biol. 2011;3. doi:10.1101/cshperspect.a002592. 10.1101/cshperspect.a002592 PMC3119911 21576252

[53] Stadtfeld M, Apostolou E, Akutsu H, Fukuda A, Follett P, Natesan S (2010). Aberrant silencing of imprinted genes on chromosome 12qF1 in mouse induced pluripotent stem cells. Nature.

[54] Mo C-F, Wu F-C, Tai K-Y, Chang W-C, Chang K-W, Kuo H-C, et al. Loss of non-coding RNA expression from the DLK1-DIO3 imprinted locus correlates with reduced neural differentiation potential in human embryonic stem cell lines. Stem Cell Res Ther. 2015;6:1. http://www.pubmedcentral.nih.gov/articlerender.fcgi?artid=4417332&tool=pmcentrez&rendertype=abstract\nhttp://www.ncbi.nlm.nih.gov/pubmed/25559585\nhttp://www.pubmedcentral.nih.gov/articlerender.fcgi?artid=PMC4417332. 10.1186/scrt535 PMC4417332 25559585

[55] Qi S, Wang Z, Li P, Wu Q, Shi T, Li J (2015). Non-germ line restoration of genomic imprinting for a small subset of imprinted genes in ubiquitin-like PHD and RING finger domain-containing 1 (Uhrf1) null mouse embryonic stem cells. J Biol Chem.

