# Supplementary figures and images for: Widespread recovery of methylation at gametic imprints in hypomethylated mouse stem cells following rescue with DNMT3A2
Source: Epigenetics Chromatin. 2016 Nov 22;9:53. doi: 10.1186/s13072-016-0104-2 (PMC5118886; doi:10.1186/s13072-016-0104-2)

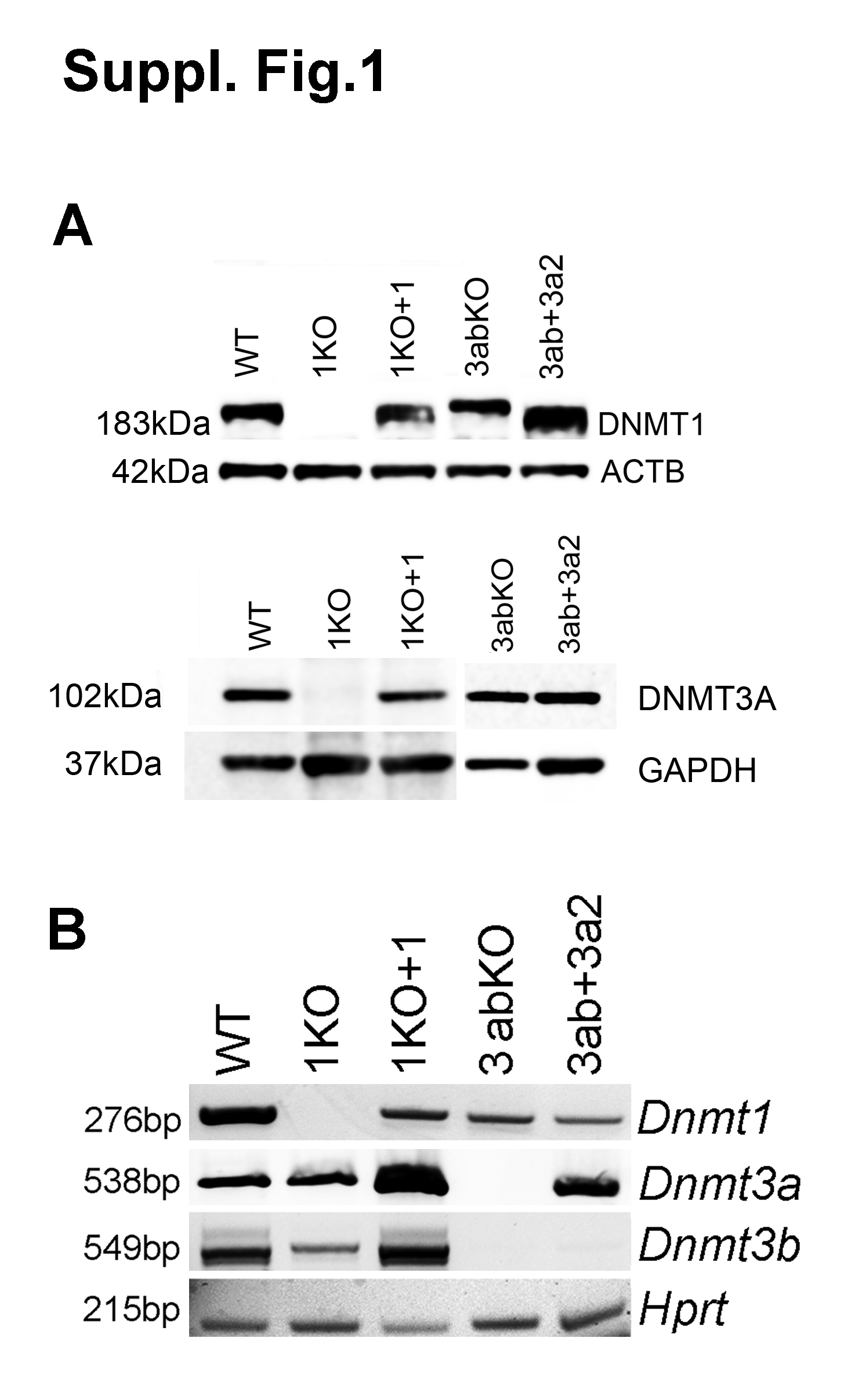

Supplement: Supplementary file 3 — Additional file 3: Figure S1. Controls to confirm the methyltransferase activities in the ESC used during the study. (A) Westerns showing the presence or absence of DNMT1 (top) or of DNMT3A2 (bottom) in the various cell lines indicated. ACTB and GAPDH are loading controls. The size of the expected protein is shown at left. A number of DNMT3B antibodies tried proved unreliable. (B) RT-PCR showing the presence or absence of transcripts for the various enzymes in the ESC used. The expected sizes are shown at left. Hprt was a loading control. [file 13072_2016_104_MOESM3_ESM.tif]
